# The glioblastoma virome across tumor and extracellular compartments

**DOI:** 10.3389/fonc.2025.1705003

**Published:** 2025-11-27

**Authors:** Mahin Ghorbani, Hamed Karimi, Farnoosh Solati, Nazanin Anbarestani, Nooshin Khoshdoozmasouleh

**Affiliations:** 1 Division of Pathology, Department of Laboratory Medicine, Karolinska Institutet, Huddinge, Sweden; 2 Operations and Business Analytics, School of Management, Universiti Sains Malaysia, Penang, Malaysia; 3 Data, AI and Analytics, Avaus Marketing Innovations, Stockholm, Sweden; 4 Department of Biology, Faculty of Basic Sciences, University of Padova, Padova, Italy; 5 Department of Cancer Medicine, University of La Trobe, Melbourne, VIC, Australia

**Keywords:** glioblastoma, virome, viral RNA, exosomes, microvesicles, ribonucleoprotein complexes, tumor microenvironment, HERV-K

## Abstract

**Background:**

Glioblastoma (GBM) is the most aggressive primary brain tumor in adults, marked by rapid progression and poor prognosis. GBM may also harbor a complex *virome*, defined here as the total repertoire of viral elements within tumors, encompassing both exogenous viruses and reactivated endogenous retroviruses. While viral signatures have been observed in other cancers, their transcriptional activity in GBM and their distribution across tumor cells and extracellular compartments remain largely unexplored.

**Methods:**

We analyzed 45 long- and small-RNA libraries from glioma stem-like cells, their extracellular fractions (exosomes, microvesicles, ribonucleoprotein complexes), and normal brain controls (BioProject PRJNA360129). Taxonomic profiling and functional annotation were performed using established pipelines (MetaPhlAn4, BLASTn, UniProtKB, InterPro), with fresh media controls to exclude background contamination. To validate discriminative power, viral RNA profiles were classified using machine learning -Random Forest models trained on nine species identified as glioblastoma-enriched by LEfSe.

**Results:**

We detected ~3.41 million viral reads, with >99% from GBM cells and extracellular compartments and <1% from normal brain. Reads spanned 36 species across 11 phyla. Nine species were consistently enriched in glioblastoma: *Saimiriine gammaherpesvirus 2, Human endogenous retrovirus K, Camelpox virus, Pestivirus A, Macacine gammaherpesvirus 5, Avian myelocytomatosis virus, Rabbit fibroma virus, Omegapapillomavirus 1*, and *Cyprinid herpesvirus 2* with genes mapping to oncogenic pathways. Random Forest models showed long-RNA profiles perfectly discriminated against tumor from normal samples (AUC = 1.0), whereas small-RNA profiles carried limited signal (AUC = 0.5). Tumor–extracellular comparisons revealed strong compartment-specific long-RNA signatures (AUC = 1.0), with partial overlap between tumor cells and exosomes in small-RNA data (AUC = 0.75).

**Conclusion:**

Glioblastoma contains a distinct viral transcriptome; long-RNA profiles show strong diagnostic power, and compartment-specific signatures suggest biomarker potential.

## Background

Glioblastoma (GB) is the most common and aggressive primary brain tumor in adults, classified as a Grade 4 malignancy by the World Health Organization (WHO). Despite advances in surgical resection, radiotherapy, and chemotherapy, patient prognosis remains poor, with a median survival of only 12–15 months (1).This dismal outcome is driven by the molecular heterogeneity of GB, its highly invasive behavior, and resistance to standard therapies (2). While numerous genetic and epigenetic alterations have been characterized (3), the complete mechanisms underlying GB pathogenesis remain incompletely understood.

Among the less-explored contributors to tumor biology, the potential role of viruses in glioblastoma has garnered increasing interest. Approximately 15% of all human cancers have a known viral component, including well-established associations with Kaposi’s sarcoma-associated herpesvirus (KSHV), Epstein–Barr virus (EBV), human papillomavirus (HPV), hepatitis B and C viruses (HBV, HCV), human T-cell lymphotropic virus type I (HTLV-1), and Merkel cell polyomavirus (MCV) (4, 5).These oncogenic viruses promote tumorigenesis through genomic integration, immune modulation, and the expression of viral proteins that subvert host signaling pathways.

Although viral DNA and RNA, such as those from Epstein–Barr virus (EBV), cytomegalovirus (CMV), SV40, and human endogenous retroviruses (HERVs), have been detected in glioblastoma tissue (6), their functional relevance remains poorly defined. Conflicting results across studies are often attributed to methodological differences, sample heterogeneity, and limitations in detection sensitivity. Notably, the reactivation of endogenous retroviruses such as HERV-K (7) within the immunosuppressive tumor microenvironment has been proposed as a potential driver of tumor progression, yet the mechanisms remain largely uncharacterized. Furthermore, metatranscriptomic approaches, capable of detecting transcriptionally active viral elements have rarely been applied to glioblastoma, leaving the role of the tumor-associated virome that encompasses the total profile of viral elements present within tumors, including both exogenous viruses and reactivated endogenous retroviruses.) significantly underexplored.

Concurrently, the field of extracellular RNA (exRNA) biology has uncovered an additional layer of molecular complexity in cancer. ExRNAs are secreted RNA species that circulate outside cells in three main forms: (1) within exosomes (<150 μm), (2) within microvesicles (200–500 nm, directly shed from the plasma membrane), and (3) as part of ribonucleoprotein (RNP) complexes. These RNA species include mRNAs, miRNAs, lncRNAs, tRNA fragments, and Y RNAs, and they play key roles in intercellular communication across both physiological and pathological states (8).

In glioblastoma, particularly in glioma stem cells (GSCs), extracellular verticals carry oncogenic molecules such as EGFRvIII, miR-21, and MGMT mRNA, which enhance tumor cell proliferation, therapeutic resistance, and survival. These EVs actively remodel the tumor microenvironment (TME) by reprogramming nearby astrocytes and microglia into tumor-supportive phenotypes, promoting immune evasion through T cell dysfunction and M2 macrophage polarization, and stimulating angiogenesis via pro-angiogenic cargo such as VEGF and miR-19b. Hypoxia further modulates the RNA composition of EVs, enhancing their invasive potential and metabolic impact (9, 10). This form of EV-mediated communication is now recognized as a defining feature of glioblastoma progression.

Despite these advances, the viral component of exRNA in glioblastoma remains almost entirely unexplored. Given the documented presence of viral nucleic acids in tumor tissue and the ability of viruses to hijack host RNA trafficking machinery (11–13), it is plausible that glioblastoma-derived extracellular compartments may selectively package viral RNAs. These RNAs could support tumor growth by modulating immune responses, enabling horizontal gene transfer, or reprogramming recipient cells. Notably, HERV-K, which is frequently reactivated in various cancers (14), may also be trafficked in extracellular vesicles (15, 16), potentially acting as a systemic oncogenic regulator.

Identifying which viruses are present within glioblastoma exRNA compartments, and determining how they are selectively distributed across exosomes, MVs, and RNPs, could reveal new insights into tumor signaling, immune modulation, and intercellular communication. Moreover, the consistent enrichment of specific viral species and viral genes may indicate active biological roles, rather than passive release, positioning these viral elements as promising candidates for diagnostic and therapeutic development.

## Materials and methods

### RNA-seq data acquisition

We analyzed long- and small-RNA sequencing libraries obtained from a publicly available dataset (BioProject: PRJNA360129) originally published by Wei et al., who mapped and quantified coding and noncoding RNA species in extracellular compartments from human glioma stem-like cells (17). These libraries were derived from patient-derived glioma stem-like cells (GSCs) and their corresponding extracellular exRNA compartments, including exosomes, microvesicles and ribonucleoprotein complexes alongside normal brain controls. The dataset comprises a total of 51 samples distributed across six groups: Cell(tumor cell), Exosome, RNP, MV, Normal, and Fresh media. The Cell, Exosome, RNP, and MV groups each contain 8 samples, evenly split between 4 longRNA and 4 smallRNA. The Normal group consists of 7 smallRNA samples derived from Neuroglia, Astrocyte, Microglia, and Neuron cell types, with an additional 6 longRNA normal cell samples selected from other databases PRJNA297760 and PRJNA975470 for comparison. The Fresh group contains both longRNA and smallRNA samples from exosomes, MVs, and RNPs, and was used as a control to account for possible contamination from environmental RNA, including viral sequences before downstream analysis.

### Read processing, quality control and viral taxonomic profiling

Raw sequencing reads were first evaluated for overall quality using FastQC (v0.72; https://www.bioinformatics.babraham.ac.uk/projects/fastqc/) to assess per-base quality scores, sequence length distribution, GC content, and the presence of adapter contamination. Adapter sequences and low-quality bases (Phred quality score < 20) were removed using Cutadapt (v5), with a minimum post-trimming read length threshold of 30 nucleotides to retain short viral fragments while ensuring accurate downstream mapping. Following quality control, taxonomic profiling of viral communities was performed using MetaPhlAn4 (v4.0.6, database updated May 2024) (18). The analysis was restricted to viral reads by applying exclusion flags (–ignore_bacteria –ignore_archaea –ignore_eukaryotes). Reads assigned to taxa with fewer than two reads or detected in fewer than 10% of samples were filtered out to minimize the risk of false positives.

Read counts for retained taxa were normalized using total sum scaling (TSS) to account for differences in sequencing depth across samples and subsequently rarefied to the minimum library size to enable direct comparisons of diversity metrics. Taxonomic assignments were generated at the family, genus, and species levels for subsequent diversity, differential abundance, and biomarker analyses.

Viral taxonomic assignment was performed using a dual-pipeline strategy combining MetaPhlAn4 and BLASTn validation against the NCBI nucleotide database. MetaPhlAn4 relies on alignment to curated clade-specific marker genes, providing high-resolution detection but limited by database completeness and representation bias. To reduce potential misclassification, we required concordant detection across both pipelines, applied stringent thresholds (≥90% sequence identity, E-value ≤0.01), prioritized multi-gene over single-gene evidence, and removed taxa also detected in fresh-media controls. This approach minimizes artifacts arising from host–virus gene homology, annotation errors, or environmental contamination. Remaining uncertainty may persist for low-read or closely related viral taxa due to inherent short-read limitations.

### Virome diversity and composition analysis

Alpha diversity (observed richness) and beta diversity (Bray–Curtis dissimilarity) metrics were calculated at the family, genus, and species levels. Beta diversity patterns were visualized using non-metric multidimensional scaling (NMDS), and statistical differences among sample groups were evaluated by PERMANOVA (vegan v2.4.3, R).

### Differential abundance and biomarker discovery

Differential enrichment of viral taxa across compartments and RNA types was performed using LEfSe (v1.1.2) (19). Taxa with p-value < 0.05 and LDA score > 2.0 were considered significant. Normalized counts were visualized as dot plots to highlight compartment-specific viral signatures.

### Validation, gene-level reconstruction, and functional annotation of viral reads

Viral reads were first obtained from MetaPhlAn4 SAM alignment files containing whole-genome shotgun (WGS) sequences, which include all viral components. The SAM files were converted to FASTA format using FASTX-Toolkit, and the resulting reads were queried with BLASTn against reference genomes of the nine viral species identified by LEfSe, using their NCBI genome accessions. For each species, mapped reads were separated and re-aligned with BLASTn against the corresponding reference genome to confirm sequence identity. The highest-scoring segment pairs (HSPs) and associated gene identifiers were recorded to validate mapping consistency. To further confirm these assignments, the viral reads were rerun through MetaPhlAn4, which again reported the same viral taxa. The Bowtie2 output from MetaPhlAn4 provided gene-level identifiers that matched those retrieved from NCBI Gene for the corresponding viral species, confirming that the recovered sequences were correctly assigned and not misclassified. Differential expression analysis of reconstructed viral genes was performed between tumor and control samples using log_2_ fold change, with adjusted *p* < 0.05 considered significant. Functional annotation with UniProtKB and InterPro classified enriched viral pathways into four major categories: DNA replication, nucleotide biosynthesis, immune modulation, and signal transduction.

### Viral biomarker prediction

To evaluate the discriminative power of viral RNA profiles across tumor and normal samples, as well as between tumor cells and their extracellular compartments, we implemented supervised machine learning using Random Forest classifiers. The analysis was restricted to nine viral species identified as glioblastoma-enriched by LEfSe. Feature matrices were derived from normalized viral read counts.

For each comparison, data were split into training (70%) and testing (30%) subsets using stratified sampling. Models were trained with 300 trees (n_estimators=300) and balanced class weights. Model performance was assessed primarily using the receiver operating characteristic area under the curve (ROC AUC), with accuracy reported as a secondary metric. Results were aggregated and used to evaluate both tumor–normal separation and the degree of similarity between tumor cells and their secreted extracellular fractions (exosomes, microvesicles, ribonucleoprotein complexes). All analyses were performed in Python 3.13.5 within a Jupyter Notebook environment using scikit-learn, pandas, numpy, and matplotlib libraries.

## Results

### Virome composition in glioblastoma and extracellular compartments

From the 45 RNA-sequencing libraries analyzed (including both long- and small-RNA fractions), a total of 3,412,910 qualified viral reads were obtained. The vast majority of reads originated from glioblastoma tumor cells and their extracellular compartments (exosomes, microvesicles, and ribonucleoprotein complexes), accounting for 3,394,234 reads (≈99.45%), whereas normal brain cell controls contributed only 18,676 reads (≈0.55%). When stratified by library type, long-RNA libraries contained 3,407,352 reads (≈99.84%), while small-RNA libraries captured 5,558 reads (≈0.16%), indicating that viral sequences were preferentially represented in longer RNA fractions. Taxonomic profiling of these reads resolved 11 phyla, 15 families, 27 genera, and 36 distinct species, highlighting the diverse and compartmentalized nature of the glioblastoma-associated virome.

A subset of viral species, including *Mule deerpox virus, Hyposoter fugitivus ichnovirus, Glypta fumiferanae ichnovirus, Dasheen mosaic virus, Cowpox virus, Aotine betaherpesvirus 1, Stx2 converting phage 1717, Vicia cryptic virus, Pandoravirus dulcis*, and *Pepper mild mottle virus*, were detected both in fresh media controls and, at low levels, in experimental samples. Given their shared presence across media and sample libraries, these viruses were interpreted as likely background contaminants and were excluded from downstream analyses to minimize false-positive associations.

**Figure 1:**
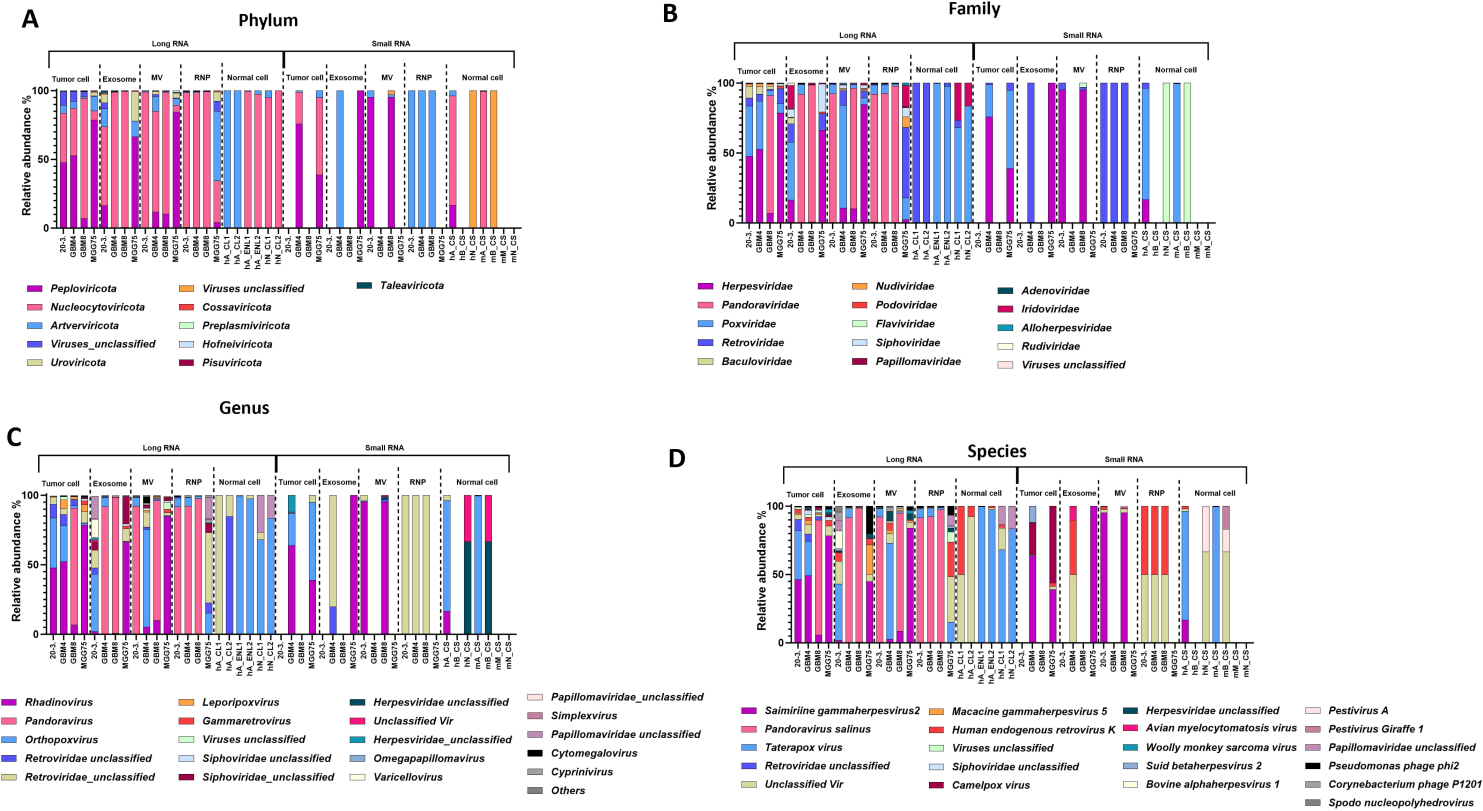
Virome composition in glioblastoma cells and extracellular RNA compartments across taxonomic levels. **(A)** Phylum-level composition. Stacked bar plots showing relative abundances of viral phyla in tumor cells, normal brain cells, and their extracellular RNA (exRNA) compartments—exosomes, microvesicles (MVs), and ribonucleoprotein complexes (RNPs), from long- and small-RNA sequencing datasets. Each bar represents a biological sample, with colors indicating different viral phyla. Read counts were normalized using total sum scaling (TSS) to account for differences in sequencing depth across samples. *Nucleocytoviricota* (pink/salmon) was dominant across tumor cells, vesicular fractions, and normal brain tissue. *Peploviricota (*magenta/purple) showed tumor-specific enrichment in exosomes and MVs but was absent or minimal in normal brain controls, suggesting selective packaging in tumor-derived extracellular compartments. Additional phyla detected include *Artverviricota, Cossaviricota, Preplasmiviricota, Hofneiviricota, Uroviricota*, and *Pisuviricota*, with variable distributions across sample types. **(B)** Family-level composition. Long-RNA libraries from tumor cells and extracellular vesicles showed enrichment in *Herpesviridae* (magenta), *Pandoraviridae* (pink/salmon)which were absent or minimal in normal brain-derived libraries. Small-RNA libraries showed lower viral diversity overall. Additional families detected include *Retroviridae, Flaviviridae, Baculoviridae, Nadiviridae, Podoviridae, Siphoviridae, Papillomaviridae, Adenoviridae, Iridoviridae, Alloherpesviridae*, and *Rudiviridae.* The family-level enrichment in tumor samples indicates specific viral lineages are associated with glioblastoma rather than representing ubiquitous background signal. **(C)** Genus-level composition. *Rhadinovirus* (magenta, a gammaherpesvirus genus) and *Pandoravirus* (pink/salmon) dominated tumor cells, exosomes, and MVs in long-RNA libraries, with minimal or no detection in normal brain samples. Additional genera detected include *Orthopoxvirus, Leporipoxvirus, Gammavirus, various Retroviridae (unclassified), Siphoviridae (unclassified), Sphiviridae*, and others. The consistent presence of *Rhadinoviru*s *and Pandoravirus* across multiple tumor-derived compartments supports biological authenticity rather than sporadic contamination. **(D)** Species-level composition. Tumor-derived long-RNA fractions were enriched for *Saimiriine gammaherpesvirus 2* (SaHV-2, magenta) and *Pandoravirus salinus* (pink/salmon), while *Taterapox virus* showed presence in normal brain cells. Additional species detected include *Human endogenous retrovirus K, Macacine gammaherpesvirus 5, Camelpox virus, Pestivirus A, Avian myelocytomatosis virus, Woolly monkey sarcoma virus, Bovine alphaherpesvirus 1, Spodoptera nucleopolyhedrovirus*, and various bacteriophages (*Pseudomonas phage phi2, Corynebacterium phage P1201*). *SaHV-2* and *Pandoravirus salinus* showed the highest abundances among tumor samples while being absent or minimal in controls. Key Finding: Viral taxonomic profiles exhibit clear tumor-specific enrichment patterns across multiple taxonomic levels (phylum, family, genus, species), with distinct compartmentalization in tumor cells and their extracellular vesicles, particularly for Herpesviridae, Pandoraviridae, and their constituent genera and species.

To define the glioblastoma-associated virome, we profiled viral RNA in normal and tumor cells and their exRNA compartments, MVs, exosomes, and RNPs, using both long- and small-RNA sequencing datasets. Viral reads were quantified with feature counts, and relative abundances were visualized across taxonomic levels using proportional abundance plots. Long-RNA libraries consistently exhibited greater viral feature richness than small-RNA libraries across all sample types. At the phylum level (**Figure 1A**), *Nucleocytoviricota* was dominant, enriched in tumor cells and vesicular fractions (exosomes, MVs) as well as in normal brain tissue. In addition, *Peploviricota* showed tumor-specific enrichment, being detected in exosomes and MVs but absent from controls. At the family level (**Figure 1B**), *Herpesviridae* and *Pandoraviridae* were abundant in long-RNA libraries from tumor cells and extracellular vesicles, yet undetectable in normal brain-derived libraries. Genus-level (**Figure 1C**) profiling revealed marked dominance of *Rhadinovirus* and *Pandoravirus* in tumor cells, exosomes, and MVs, with no detection in any control datasets. Species-level (**Figure 1D**) analysis confirmed this compartmentalization: tumor-derived long-RNA fractions were enriched for *Saimiriine gammaherpesvirus 2* and *Pandoravirus salinus*, whereas *Taterapox virus* predominated in normal brain cells. Across tumor-derived compartments, virome profiles were broadly similar but displayed fraction-specific enrichment patterns. Small-RNA libraries exhibited lower viral diversity, capturing only a subset of taxa observed in the long-RNA data, consistent with selective processing, stabilization, or preferential retention of specific viral RNAs within smaller RNA fractions.

### Alpha and beta diversity of the glioblastoma virome

**Figure 2:**
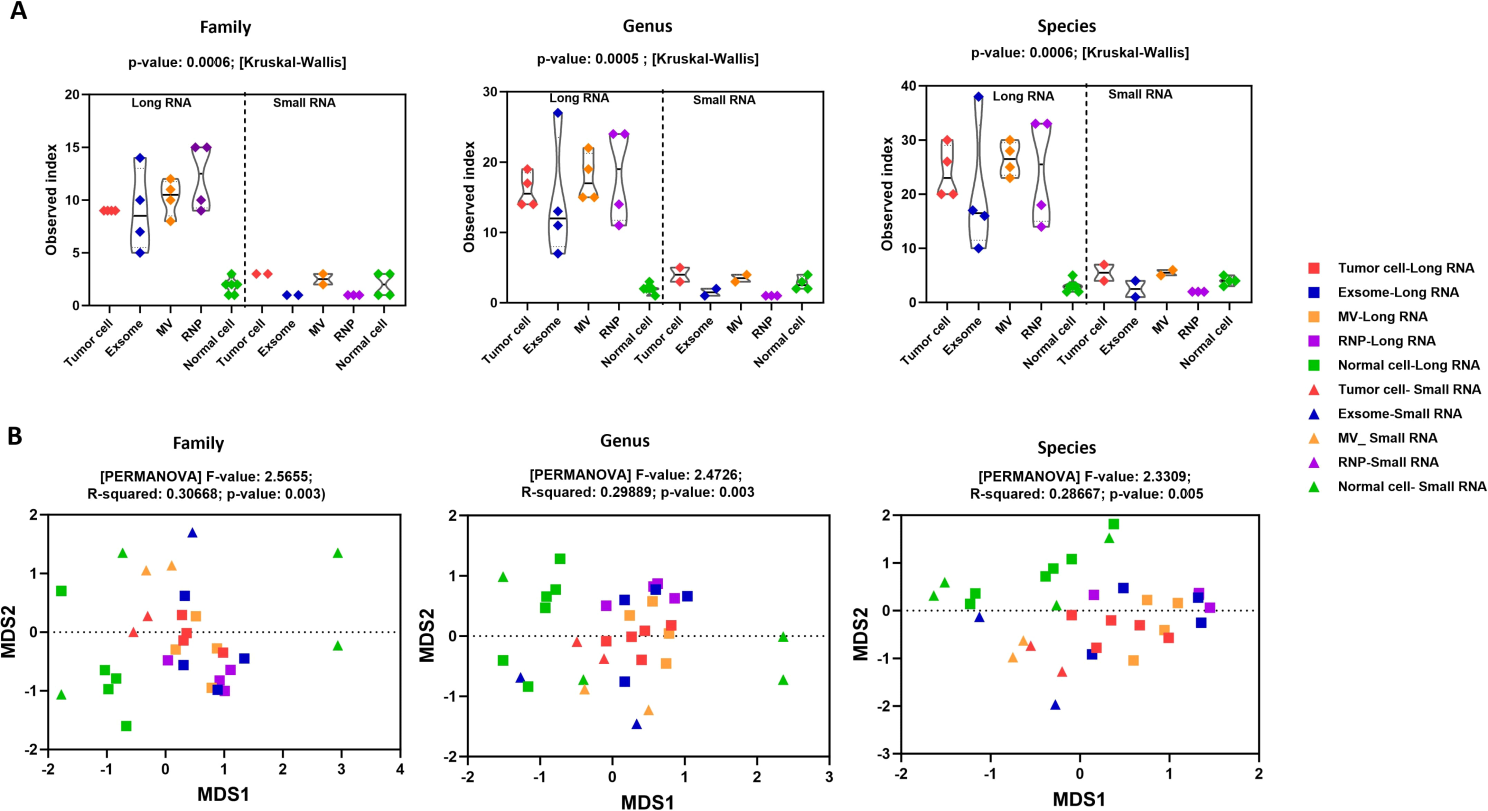
Alpha and beta diversity of the glioblastoma virome across RNA fractions and sample types. **(A)** Alpha diversity analysis. Violin plots with overlaid box plots showing observed richness (Observed index, representing the number of distinct viral taxa detected) compared across long-RNA and small-RNA fractions at the family, genus, and species levels. Each point represents an individual biological sample. Colors indicate sample types: red = tumor cell-long RNA, blue = exosome-long RNA, orange = MV-long RNA, purple = RNP-long RNA, green = normal cell-long RNA (squares for long RNA); and corresponding triangles for small-RNA fractions. Long-RNA fractions (left side of each panel) showed significantly higher viral diversity than small-RNA fractions (right side) across all taxonomic levels (family: p = 0.0006; genus: p = 0.0005; species: p = 0.0006; Kruskal–Wallis test). Tumor cells and their extracellular compartments (exosomes, microvesicles, and RNPs) consistently exhibited greater richness than normal brain cell samples, as indicated by higher median values (box plot horizontal lines) and broader distributions (violin width). Normal brain samples (green) showed the lowest viral diversity, particularly in long-RNA fractions. **(B)** Beta diversity analysis. Non-metric multidimensional scaling (NMDS) ordination plots based on Bray–Curtis dissimilarity matrices at the family, genus, and species levels. Each point represents a biological sample, with colors and shapes indicating sample type as in panel **(A)** Samples that cluster closer together have more similar viral community compositions, while distant samples differ in their viral profiles. PERMANOVA (permutational multivariate analysis of variance) testing confirmed significant differences in viral community composition between tumor-derived and normal samples at all taxonomic levels (family: F = 2.5655, R² = 0.3067, p = 0.003; genus: F = 2.4726, R² = 0.2989, p = 0.003; species: F = 2.3309, R² = 0.2867, p = 0.005). Note the separation pattern: normal brain samples (green triangles and squares) cluster in the upper right quadrant, while tumor-associated samples (red, blue, orange, purple) distribute across different regions, indicating distinct viral communities. Long-RNA samples (squares) show clearer separation than small-RNA samples (triangles). Key Finding: Glioblastoma samples exhibit both significantly higher viral diversity (alpha diversity) and distinct viral community composition (beta diversity) compared to normal brain tissue. Long-RNA fractions capture substantially more viral diversity than small-RNA fractions, and tumor-derived compartments (cells, exosomes, MVs, RNPs) share similar viral profiles that differ markedly from normal brain controls.

Alpha diversity analysis revealed statistically significant differences in viral richness across sample types and RNA fractions. At all examined taxonomic levels, family, genus, and species (**Figure 2A**), long-RNA fractions exhibited markedly higher viral diversity than their small-RNA counterparts (family: p = 0.0006; genus: p = 0.0005; species: p = 0.0006). Tumor cells and their associated extracellular compartments (exosomes, microvesicles, and RNPs) consistently displayed greater richness than normal brain cell samples, indicating selective viral enrichment within glioblastoma-derived RNA populations. Beta diversity analysis performed using non-metric multidimensional scaling (NMDS) based on Bray–Curtis dissimilarity, demonstrated clear separation between tumor-derived and normal samples across all taxonomic levels (**Figure 2B**). PERMANOVA testing confirmed statistically significant differences in viral community composition at the family (F = 2.57, R² = 0.307, p = 0.003), genus (F = 2.47, R² = 0.299, p = 0.003), and species levels (F = 2.33, R² = 0.287, p = 0.005), reflecting consistent divergence in virome structure between tumor and normal brain compartments.

### Distinct viral signatures differentiate glioblastoma from normal brain

**Figure 3:**
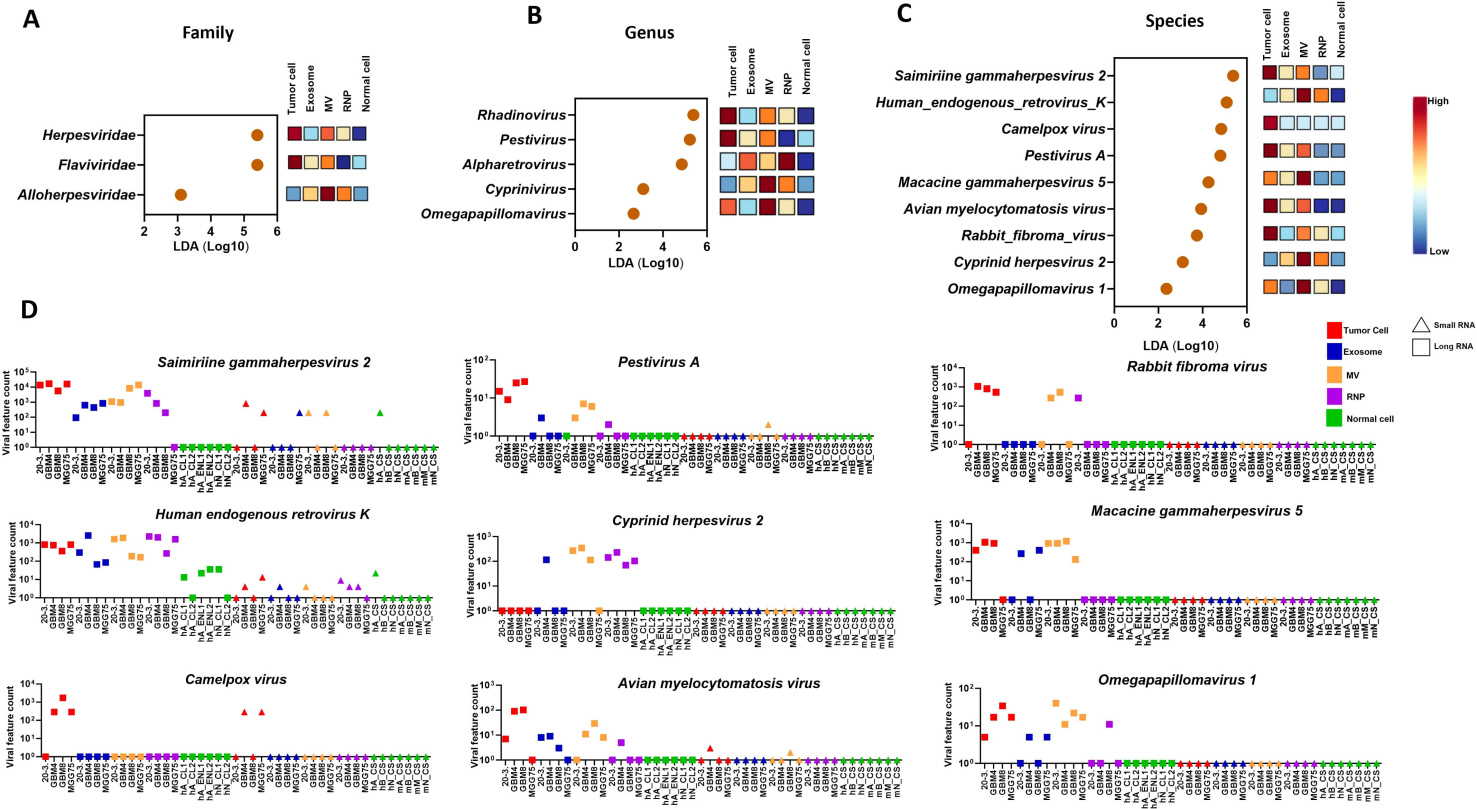
Differentially enriched viral taxa in glioblastoma compared to normal brain tissue identified by LEfSe analysis. Linear discriminant analysis (LDA) effect size (LEfSe) was used to identify viral taxa that are significantly and consistently enriched in tumor versus normal samples. LEfSe combines statistical significance testing (Kruskal-Wallis test, p < 0.05) with biological relevance assessment (LDA score > 2.0) to identify biomarkers. The position of each orange dot along the x-axis indicates the magnitude of enrichment (LDA score on log10 scale). **(A)** Family-level enrichment. Dot plot showing viral families significantly enriched in glioblastoma. Each orange dot represents a viral family, with the LDA score indicating the strength of enrichment. Colored squares to the right show relative abundance patterns across sample types (red/dark = high, blue = low): tumor cell, exosome, MV, RNP, and normal cell. *Herpesviridae* showed the strongest enrichment (LDA score ~6), followed by *Flaviviridae* and *Alloherpesviridae.* These families were highly abundant in tumor-derived compartments (red/orange squares) while being depleted in normal brain (blue squares).**(B)** Genus-level enrichment. Five genera were significantly enriched in glioblastoma: *Rhadinovirus (*highest LDA score ~6), *Pestivirus, Alpharetrovirus, Cyprinivirus*, and *Omegapapillomavirus.* The heatmap squares show consistent enrichment across tumor compartments (red/orange) and depletion in normal controls (blue).**(C)** Species-level enrichment. Nine viral species were significantly enriched in tumor-derived RNA, listed from top to bottom*: Saimiriine gammaherpesvirus 2* (SaHV-2, LDA score ~6), *Human endogenous retrovirus K (HERV-K), Camelpox virus, Pestivirus A, Macacine gammaherpesvirus 5 (MaHV-5), Avian myelocytomatosis virus, Rabbit fibroma virus, Cyprinid herpesvirus 2, and Omegapapillomavirus 1*. The orange dots indicate LDA scores, while heatmap squares show abundance patterns across sample types. **(D)** Viral feature counts. Scatter plots showing absolute viral read counts (log10 scale) for each of the nine enriched species across individual samples. Colors indicate sample types: red = tumor cell, blue = exosome, orange = MV, purple = RNP, green = normal cell. Shapes indicate: filled squares = long-RNA, triangles = small-RNA. Tumor samples show 10^2^ to 10^4^ reads per sample, while normal samples show 10^0^ to 10^1^ (minimal/absent). The top three most abundant species are *Saimiriine gammaherpesvirus 2* (>10^4^ reads), *HERV-K* (~10^4^ reads), and *Macacine gammaherpesvirus 5* (~10³ reads). Legend indicates filled squares = long-RNA, open squares = small-RNA. Key Finding: Nine viral species show statistically significant enrichment in glioblastoma across cellular and extracellular compartments, with dramatic depletion in normal brain tissue, establishing them as candidate tumor-associated viral biomarkers.

LEfSe analysis (LDA > 0.2, p < 0.05) revealed marked enrichment and depletion of viral taxa across glioblastoma-derived samples compared to normal brain tissue. At the family level (**Figure 3A**), *Herpesviridae, Flaviviridae*, and A*lloherpesviridae* were enriched in tumor cells, exosomes, MVs, and RNPs, but depleted in normal brain compartments. Genus-level analysis (**Figure 3B**) showed enrichment of *Rhadinovirus, Pestivirus, Alpharetrovirus, Cyprinivirus*, and *Omegapapillomavirus* in glioblastoma fractions, with corresponding depletion in normal controls.

At the species level (**Figure 3C**), *Saimiriine gammaherpesvirus 2, Human endogenous retrovirus K, Camelpox virus, Pestivirus A, Macacine gammaherpesvirus 5, Avian myelocytomatosis virus, Rabbit fibroma virus, Omegapapillomavirus 1*, and *Cyprinid herpesvirus 2* were enriched in tumor-derived RNA, particularly within tumor cells, exosomes, and MVs, and consistently depleted in normal brain samples.

These patterns were supported by feature count profiles, (**Figure 3D**). Which demonstrated elevated viral loads in tumor-associated compartments. Overall, the data reveal a distinct virome in glioblastoma, characterized by the selective enrichment of specific viral taxa and their depletion in normal brain tissue.

### Long RNA viral profiles accurately distinguish tumor from normal and define compartment-specific signatures

**Figure 4:**
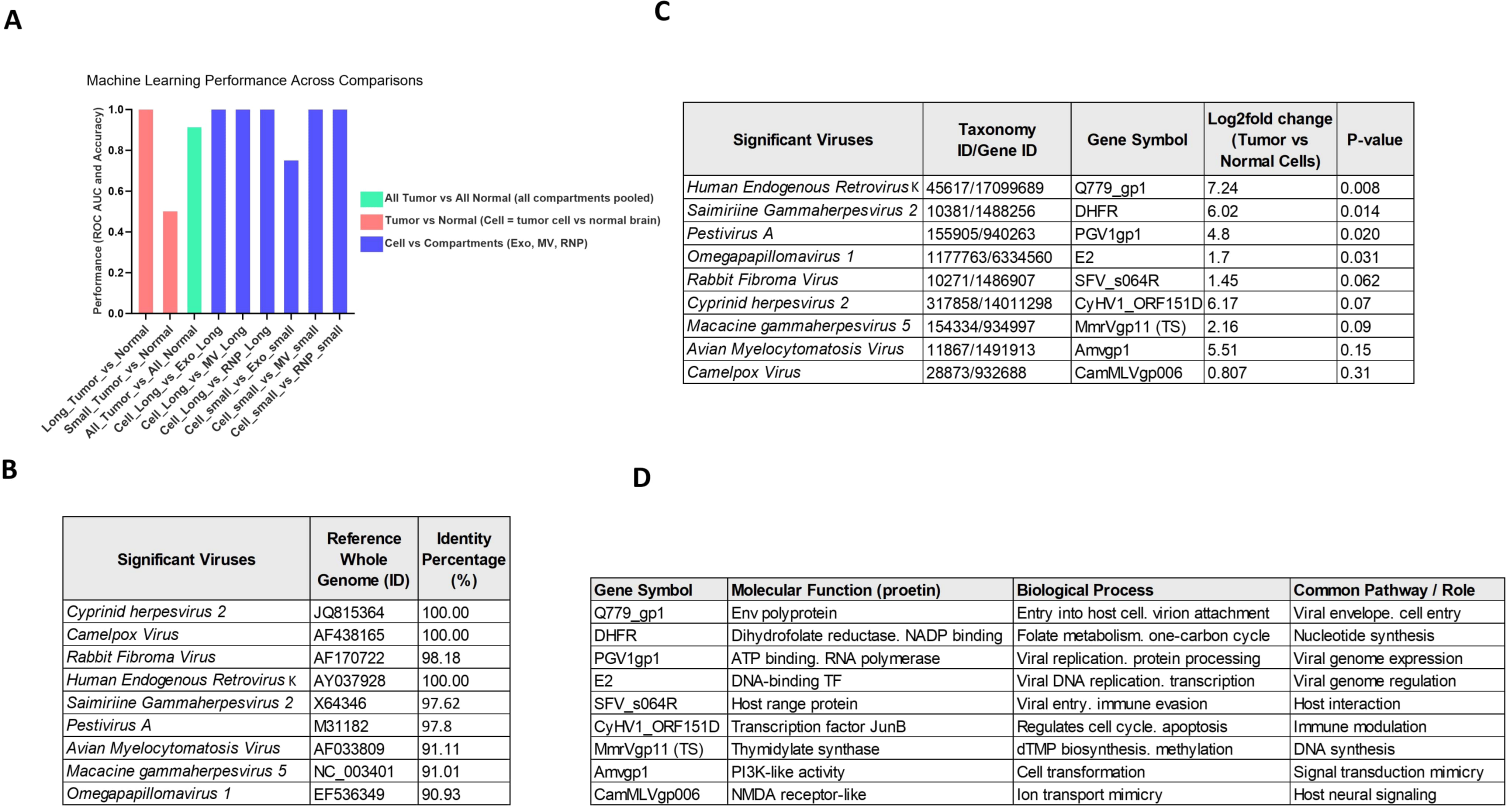
Enriched viral sequences and genes in glioblastoma and their diagnostic potential. **(A)** Machine learning classification performance. Random Forest models were trained using the nine viral species identified as significantly enriched in glioblastoma by LEfSe analysis. The performance is reported as ROC AUC (Area Under the Receiver Operating Characteristic Curve). Long-RNA expression profiles achieved perfect discrimination between tumor and normal brain samples (ROC AUC = 1.0), as well as between tumor cells and their extracellular RNA compartments (exosomes, microvesicles, and RNP complexes; ROC AUC = 1.0), indicating strong compartment-specific viral RNA signatures. In contrast, small-RNA profiles contained limited discriminatory information (Tumor vs Normal: ROC AUC = 0.50), but showed partial overlap between tumor cells and exosomes (ROC AUC = 0.75), suggesting selective packaging of small viral RNAs into extracellular vesicles. **(B)** Sequence identity validation. Viral reads detected in glioblastoma samples were validated by BLASTn alignment against reference genomes in the NCBI nucleotide (nt) database. All nine enriched viral species exhibited high nucleotide sequence identity (≈90–100%) to their nearest reference genomes, with very low E-values (≤1e-13). The highest scoring HSP (High-Scoring Pair) per species is shown. These high-confidence alignments support the authenticity of the viral signal and rule out spurious annotation or sequencing artifacts. **(C)** Viral gene annotation and differential expression. Viral transcripts were mapped to gene models, and differential expression was quantified between tumor cells and normal brain cells. Several genes were significantly overexpressed in glioblastoma (adjusted p < 0.05), including Q779_gp1 from *Human Endogenous Retrovirus K* (log_2_FC = 7.24), DHFR from *Saimiriine gammaherpesvirus 2* (log_2_FC = 6.02), and PGV1gp1 from *Pestivirus A* (log_2_FC = 4.80). Additional genes, such as *Omegapapillomavirus* E2 and *Macacine gammaherpesvirus* MmrVgp11, showed moderate upregulation. These transcripts represent active viral gene expression, rather than passive genomic remnants. **(D)** Functional roles of enriched viral genes. Annotated viral genes were assigned to molecular functions and biological processes. Overexpressed viral proteins mapped to key oncogenic pathway categories, including: viral entry and membrane fusion (e.g., envelope polyprotein Q779_gp1),nucleotide and DNA synthesis (DHFR, thymidylate synthase-like MmrVgp11),viral genome replication and transcription control (PGV1gp1, E2), host-pathway mimicry and cell signaling (Amvgp1 with PI3K-like activity; CamMLVgp006 with NMDA receptor-like function).These functional profiles suggest that viral transcripts in glioblastoma may participate in metabolic rewiring, transcriptional regulation, immune evasion, and signaling modulation. Key Finding: Viral RNA signatures in glioblastoma are robust, biologically consistent across cellular and extracellular compartments, diagnostically discriminative, and include overexpressed viral genes with potential functional relevance to tumor biology. However, lower-abundance viral species may require additional experimental validation to confirm biological relevance, and the small sample size should be considered when interpreting machine-learning performance metrics (ROC AUC values), which will benefit from validation in larger independent cohorts.

Random Forest models demonstrated distinct performance across tumor–normal and cell–extracellular comparisons (**Figure 4A**). Long tumors were perfectly distinguished from normal samples (ROC AUC = 1.0, Accuracy = 1.0), and pooled tumor samples also achieved high discrimination (ROC AUC = 0.91, Accuracy = 0.86). Although small tumors showed weaker separation from normal samples (ROC AUC = 0.5, Accuracy = 0.75), this likely reflects limited discriminative signal and class imbalance rather than the absence of biological differences.

Comparisons between tumor cells and their extracellular compartments yielded uniformly high ROC AUC values (1.0 for most contrasts), indicating strong underlying separation capacity. Accuracy values were consistently more modest (0.67 across most tasks), which can be attributed to dataset imbalance and overlapping profiles. The only exception was *Cell_small vs Exo_small*, which showed reduced separation (ROC AUC = 0.75).

Overall, these results underscore the robust ability of viral RNA profiles to distinguish tumor from normal samples, particularly in long and pooled tumors, while also revealing a high degree of molecular similarity between tumor cells and their extracellular vesicle-associated fractions.

### Transcriptionally active viral signatures in glioblastoma and their diagnostic potential

BLAST confirmed the presence of multiple viral signatures with high local sequence similarity to known viral genomes. Several reads aligned perfectly (100% identity) to regions of *Camelpox virus, Cyprinid herpesvirus 2, and Human endogenous retrovirus K (HERV-K).* Other taxa, including *Pestivirus A, Rabbit fibroma virus*, and *Saimiriine gammaherpesvirus* 2, showed strong but not perfect similarity (97–98%). Lower, yet still significant, matches (~91%) were found for *Avian myelocytomatosis virus* and *Macacine gammaherpesvirus 5*.These matches were supported by extremely low E-values (≤ 1e-13), indicating that the observed alignments are highly unlikely to be random. Multiple independent reads aligned to similar regions within the same viral genomes, providing additional support for their presence (**Figure 4B**).

Gene-level analysis of tumor cells versus normal cells revealed multiple overexpressed viral genes. The most significantly upregulated was Q779_gp1 *from Human Endogenous Retrovirus* K (log_2_FC = 7.24, p = 0.008), followed by DHFR from *Saimiriine gammaherpesvirus 2* (log_2_FC = 6.02, p = 0.014) and PGV1gp1 from *Pestivirus A* (log_2_FC = 4.80, p = 0.020). The E2 gene from *Omegapapillomavirus 1* also showed moderate overexpression (log_2_FC = 1.70, p = 0.031). Additional genes, including SFV_s064R from *Rabbit Fibroma Virus* (log_2_FC = 1.45, p = 0.062) and CyHV1_ORF151D from *Cyprinid herpesvirus 2* (log_2_FC = 6.17, p = 0.070), exhibited notable expression increases despite not meeting conventional significance thresholds.

Moderately overexpressed but lower-significance genes included MmrVgp11 (TS) from *Macacine gammaherpesvirus 5 (*log_2_FC = 2.16, p = 0.090) and Amvgp1 from *Avian Myelocytomatosis Virus* (log_2_FC = 5.51, p = 0.150). The smallest observed change was for CamMLVgp006 from *Camelpox Virus* (log_2_FC = 0.807, p = 0.310). Based on statistical significance and expression magnitude, HERV-K, *Saimiriine gammaherpesvirus 2*, and *Pestivirus A* emerged as the strongest candidates for further functional investigation (**Figure 4C**).

Functional annotation (**Figure 4D**) linked these overexpressed viral genes to oncogenic pathways, including viral entry, genome replication, nucleotide biosynthesis, transcriptional regulation, and immune modulation. Specific functional roles encompassed viral envelope proteins, PI3K-like transformation activity, transcription factors, and ion channel mimicry.

Collectively, these findings show that glioblastoma harbors a transcriptionally active virome enriched in viral genes that are overexpressed in tumor cells compared with normal cells, many of which have functional roles in immune evasion, oncogenic signaling, and intercellular communication. Such viral elements represent promising candidates for biomarker development and targeted therapeutic strategies in GBM.

## Discussion

This study suggests that glioblastoma harbors a diverse and transcriptionally active virome, with enrichment particularly evident in extracellular RNA (exRNA) compartments such as exosomes, microvesicles (MVs), and ribonucleoprotein complexes (RNPs). Viral species were largely absent or depleted in normal brain controls, and their alignment with known viral genomes supports their likely biological origin rather than technical artifact. The structured localization of these transcripts across distinct compartments, together with their annotated gene functions, indicates potential biological relevance to tumor processes.

Our metatranscriptomic profiling revealed multiple viral genes with sequence similarity to proteins involved in oncogenic signaling and host-pathway mimicry. These originated from both human-associated and non-human viruses and appeared selectively packaged within tumor-derived compartments, suggesting coordinated viral–host interactions that may contribute to glioblastoma biology through molecular mimicry, pathway co-option, or expression of anciently integrated viral elements.

Among the nine viral species identified, we focused in detail on *Saimiriine gammaherpesvirus 2* and *human endogenous retrovirus K (HERV-K)*, which demonstrated the strongest biological evidence and the highest cumulative viral read counts (exceeding 100,000). These showed the most consistent and reproducible expression patterns across samples. The remaining viral signals were of lower or variable significance, likely reflecting limited sample size or underlying biological heterogeneity. They are reported for completeness but will require additional validation before firm conclusions can be drawn.

We detected DHFR from *Saimiriine gammaherpesvirus 2* in GBM-derived compartments. DHFR is a key enzyme in nucleotide biosynthesis and a known target of methotrexate; its overexpression supports self-renewal in glioma stem cells and contributes to therapy resistance (20, 21). Its presence in extracellular vesicles raises the possibility of virally encoded metabolic enhancement. This suggests that virally derived DHFR may mimic or enhance tumor cell metabolic pathways.

We also detected HERV-K (HML-2) env (Q779_gp1) selectively in exosomes and RNPs. HERV-K env promotes glioma cell survival, ERK/AKT activation, and immune evasion (22).Recent studies further show that HERV-K env increases chemosensitivity in ovarian cancer by inhibiting the NF-κB/P-glycoprotein axis (23), and contributes to glioblastoma stem cell plasticity via OCT4 regulation (24).

Although our study is not yet supported by experimental validation, previous research has provided partial empirical evidence for some of the viral elements identified and their functional relevance in glioblastoma and other cancers. The role of human endogenous retrovirus K (HERV-K, HML-2) in glioblastoma has been experimentally demonstrated: CRISPR interference targeting HML-2 LTR5Hs elements in patient-derived glioblastoma neurospheres led to marked reductions in neurosphere formation, OCT4 and Nestin expression, and tumorigenicity in intracranial xenografts, resulting in prolonged survival. These findings indicate that HERV-K (HML-2) actively drives the glioblastoma stem-cell phenotype rather than functioning as a passive transcriptional byproduct (24).

For non-human viral sequences, particularly *Saimiriine gammaherpesvirus 2* (SaHV-2), while their biological significance in human glioblastoma remains to be validated, prior studies establish a precedent for functional activity of viral homologs in mammalian system.SaHV-2, a *gammaherpesvirus* naturally found in squirrel monkeys, has been extensively studied as a model of viral oncogenesis due to its ability to induce lymphoproliferative disease in non-natural primate hosts (25, 26).Notably, this virus encodes dihydrofolate reductase (DHFR) gene that shares substantial sequence identity with its mammalian counterpart and demonstrates enzymatic function in human cells. Early molecular characterization revealed that the viral DHFR can complement loss of host DHFR activity under selective pressure, indicating that it is not merely a genomic remnant but retains catalytic competence (26, 27). This observation is potentially relevant to glioblastoma biology, as recent work has shown that targeting the folate pathway through DHFR inhibition impairs the self-renewal capacity of glioma-initiating cells (21).Therefore, our detection of SaHV-2 DHFR-homologous transcripts in glioblastoma samples, while unexpected, suggests a hypothesis worth testing: that viral-derived metabolic genes may contribute to the altered folate metabolism and antifolate resistance characteristic of aggressive gliomas (28).Direct experimental validation, including CRISPR-based perturbation and functional rescue experiments, will be necessary to establish whether these transcripts represent active functional elements or incidental sequence homology.

Several additional viral transcripts were detected but exhibited markedly lower viral feature counts compared with *Saimiriine gammaherpesvirus 2* and HERV-K. These includeese include E2 transcripts from *Omegapapillomavirus* 1 with moderate enrichment in GBM compartments (29–31). *Cyprinid herpesvirus 1* (CyHV-1) ORF151D showing similarity to human JunB, and three viral proteins (32), MmrVgp11 (thymidylate synthase–like), AmvGp1 (PI3K-like), and CamMLVgp006 (NMDA receptor–like)—that display sequence homology to host oncogenic proteins (33–35). Because their abundance and statistical significance were lower, these findings are reported without detailed interpretation. They remain speculative and will require further validation before any functional conclusions can be established.

Taken together, these observations support a working hypothesis—derived from integrative bioinformatic analyses—that glioblastoma harbors a structured and transcriptionally active virome. The identified viral genes appear to mimic or intersect with core cellular pathways regulating metabolism, stemness, and immune modulation. While these findings are compelling, they remain based on computational evidence rather than direct experimental proof and should therefore be regarded as hypothesis-generating. Future mechanistic studies employing CRISPR perturbation, functional rescue, and metabolic flux assays will be essential to confirm the biological activity and significance of these viral elements in glioblastoma.

Several identified viruses; *Saimiriine gammaherpesvirus 2, Macacine gammaherpesvirus 5*, and *Cyprinid herpesvirus 2*,are of zoonotic or non-human origin. Their gene expression in GBM exRNA could reflect ancient integration events, horizontal transfer, or molecular mimicry (36–39). Horizontal gene transfer (HGT) between viruses and hosts, as well as among different virus lineages, is a well-established evolutionary process. Recent studies show that many animal viruses, especially large DNA viruses such as *herpesviruses* and *poxviruses*, frequently acquire genes from their hosts. These captured genes often encode metabolic enzymes, immune-modulatory proteins, or signaling factors, which can help the virus evade immune responses or control host cell functions (36).Similarly, bidirectional gene transfer between eukaryotes and viruses has been documented across diverse viral families, enabling viruses to co-opt host cellular machinery and, conversely, allowing host genomes to capture and repurpose viral genes (37).In some cases, viral genes encoding host protein mimics facilitate immune evasion through molecular mimicry, whereby viral proteins structurally resemble host immune regulators to subvert antiviral responses (38).

The *Saimiriine gammaherpesvirus* DHFR shares 83% amino acid identity with human DHFR and lacks introns, indicating acquisition through reverse transcription of mammalian mRNA (26)providing established precedent for functional metabolic gene transfer in *gammaherpesviruses*. However, we acknowledge that definitive confirmation of integration and biological relevance, particularly for non-human viral sequences, will require further validation such as whole-genome sequencing, PCR from genomic DNA, and spatial localization assays. Furthermore, HERV-K is a well-characterized endogenous retrovirus that integrated into the ancestral primate genome millions of years ago and is now fixed in the human germline. Approximately 8% of the human genome is composed of human endogenous retroviruses (40). While most HERV elements are transcriptionally silenced, HERV-K retains coding capacity and undergoes reactivation in multiple cancers (41), including glioblastoma, where it has been experimentally validated to promote stem cell maintenance and tumorigenesis (24).The detection of HERV-K overexpression in our dataset is therefore consistent with established biology rather than technical artifact, providing biological support for the authenticity of viral signatures in our data. The differential localization of viral transcripts—such as HERV-K env in exosomes and SaHV-2 DHFR in microvesicles—suggests selective packaging, potentially mediated by RNA-sorting proteins like hnRNPA2B1 and YBX1 (42). This targeted enrichment supports a possible role for viral RNAs in intercellular communication, metabolic adaptation, and immune modulation within the glioblastoma microenvironment.

Building on this, it is noteworthy that several well-established human oncogenic viruses share clear evolutionary relationships with viruses circulating in non-human hosts, illustrating that cross-species transmission can give rise to cancer-associated infections in humans. For example, Kaposi’s sarcoma–associated herpesvirus (KSHV/HHV-8) belongs to the *Rhadinovirus* genus and is closely related to *gammaherpesviruses* endemic to Old World primates, including macaques (43).Likewise, Epstein–Barr virus (EBV), the cause of Burkitt lymphoma and nasopharyngeal carcinoma, shares ancestry with lymphocryptoviruses found in non-human primates (44). High-risk human papillomaviruses (e.g., HPV-16 and HPV-18) cluster within the *Alphapapillomavirus* genus alongside papillomaviruses that infect other primates, supporting a model of host co-divergence with episodes of ancient host switching, rather than a purely human-restricted origin This evolutionary relationship reflects conserved mechanisms of epithelial niche adaptation, latency maintenance, and immune evasion that facilitate persistent infection and oncogenic potential (45).In addition, several human tumor-associated viruses show evidence of historical zoonotic transmission (46).Human T-cell leukemia virus type 1 (HTLV-1), the established cause of adult T-cell leukemia/lymphoma, arose from cross-species transmission of *simian deltaretroviruses (*47, 48).Merkel cell polyomavirus, the driver of Merkel cell carcinoma (49), shares a common ancestor with *polyomaviruses* infecting small mammals (50), reflecting a broader evolutionary pool of oncogenic polyomaviruses. The relationship between *Simian Virus 40* (SV40) and human tumors remains debated; while SV40 large T-antigen is oncogenic in experimental systems, its causal role in human cancers has not been definitively established, and therefore we discuss it here as an example of viral oncogenic potential rather than confirmed human tumor etiology (51, 52).

Because humans and non-human primates share 75–98.5% genetic homology and highly similar cellular and immune environments, their viruses often display overlapping tropisms, latency mechanisms, and immune evasion strategies (46, 53).Endogenous retroviral elements such as HERV-K remain transcriptionally active in gliomas, suggesting that the tumor virome may represent both ancient viral integrations and context-dependent reactivation within the transformed cellular environment. Taken together, the detection of non-human–annotated viral signatures in glioblastoma should not be regarded as incidental. These may represent latent reactivation, ancestral viral remnants, molecular mimicry, or selective viral transcript retention within tumor-associated pathways. Nonetheless, we emphasize that species-level assignments from short-read sequencing carry inherent uncertainty; thus, these interpretations remain hypothesis-generating. Future validation using orthogonal methods—such as RNA-FISH, targeted RT-qPCR, long-read sequencing, or phylogenomic reconstruction—will be critical to determine their biological presence and functional significance.

Our identification of tumor-specific viral RNA signatures in extracellular compartments (exosomes, microvesicles, RNPs) aligns with the growing clinical interest in liquid biopsy approaches for glioblastoma diagnosis and monitoring. Extracellular vesicles from glioblastoma patients carry tumor-specific molecular cargo, including mutant EGFRvIII, tumor-associated miRNAs, and oncogenic mRNAs, that can be detected in peripheral blood and cerebrospinal fluid (54–56).

These circulating biomarkers have shown clinical promise for non-invasive tumor detection, molecular subtyping, and treatment response monitoring.

Recent clinical studies have validated the feasibility of EV-based diagnostics in glioblastoma cohorts. For example, Osti et al. (2019) demonstrated that plasma extracellular vesicle profiles can distinguish glioblastoma patients from healthy controls with high sensitivity and specificity (54)while Hallal et al. (57) identified prognostically significant protein biomarkers in neurosurgical aspirate-derived EVs. Small noncoding RNA signatures in serum exosomes have been proposed as diagnostic tools and circulating cell-free DNA analysis has shown diagnostic and prognostic value in multicenter glioma cohorts (58–60).

Our finding that viral RNA species—particularly HERV-K, Saimiriine gammaherpesvirus 2, and Macacine gammaherpesvirus 5—are enriched in tumor-derived extracellular vesicles raises the possibility that viral signatures could serve as complementary or orthogonal biomarkers to existing EV-based diagnostic panels. The significant discrimination achieved by long-RNA viral profiles in our machine learning models suggests significant diagnostic potential, though this must be validated in larger cohort.

Importantly, the clinical translation of EV-based viral biomarkers would require: (1) validation in plasma or CSF from glioblastoma patients versus healthy controls and patients with other brain pathologies, (2) assessment of biomarker stability during sample collection and processing, (3) correlation with tumor burden, molecular subtype, and clinical outcomes, (4) standardization of EV isolation and viral RNA detection protocols across clinical laboratories, and (5) prospective validation in multicenter cohorts. Recent advances in microfluidic chip-based EV analysis (56) and digital PCR methods for rare transcript detection (61)provide technical platforms that could be adapted for clinical viral RNA profiling.

Given the immunosuppressive role of glioblastoma-derived EVs (62) and the established function of HERV-K in maintaining cancer stem cell populations (24) viral RNA cargo within extracellular vesicles may represent not only diagnostic markers but also functional mediators of tumor progression and immune evasion. This dual role—biomarker and effector—warrants further investigation in patient-derived samples and functional models.

Collectively, these findings indicate that the glioblastoma extracellular RNA landscape includes a distinct and organized viral component, integrated into the tumor’s molecular network. Although the current data are derived from bioinformatic analyses and require experimental confirmation, the observed patterns point to a potentially meaningful layer of tumor complexity with implications for glioblastoma progression, cellular communication, and therapeutic targeting.

Interpretation of non-human viral signatures is inherently constrained by the dependence on reference databases and the limitations of short-read sequencing, particularly where host–virus homology, integration events, or molecular mimicry obscure species-level resolution. Nevertheless, our dual-pipeline validation, stringent quality control, and compartment-specific reproducibility provide strong support for the authenticity of these viral signals. We acknowledge that residual uncertainty may remain for low-abundance or highly conserved viral sequences, which should be confirmed using orthogonal methods such as long-read sequencing, *de novo* assembly, phylogenetic reconstruction, targeted PCR/ddPCR, or RNA-FISH.

We acknowledge the inherent limitations of using publicly available RNA-seq datasets, including modest sample size, variable processing protocols, and cohort heterogeneity. To address these challenges, we implemented a unified analysis pipeline with rigorous quality control, reagent-free negative controls, and stringent contamination filtering to minimize technical bias. We prioritized the most consistently detected and biologically relevant viral species (Saimiriine gammaherpesvirus 2 and HERV-K), while excluding low-confidence or sporadic signals pending further validation. Although the Random Forest model achieved an AUC = 1.0 when distinguishing glioblastoma from normal samples, this result should be interpreted with caution. Such perfect separation may partly reflect the modest sample size, well-defined class boundaries, and potential batch effects between studies, all of which can inflate classifier accuracy. While the observed performance likely captures genuine biological differences in viral transcriptomes, future validation using larger, prospectively collected cohorts and independent datasets will be essential to confirm reproducibility and rule out model overfitting.

## Conclusion

In summary, this study provides the first comprehensive characterization of the glioblastoma virome across cellular and extracellular compartments, revealing tumor-specific viral signatures with potential functional and diagnostic relevance. While definitive validation remains essential, these findings establish a hypothesis-generating foundation for understanding the viral landscape of glioblastoma and its potential contributions to tumor biology, immune evasion, and intercellular communication. Future work involving larger patient cohorts, *in vivo* functional studies, and investigation of viral RNAs in biofluids will be important for clarifying their role and translational relevance.

## Data Availability

The original RNA-seq data from the referenced study are available in the NCBI SRA under BioProject PRJNA360129. The newly generated viral data and analyses from this study are provided as supplementary files.
